# Polyelectrolytes Enabled Reduced Graphite Oxide Water Dispersions: Effects of the Structure, Molecular Weight, and Charge Density

**DOI:** 10.3390/polym14194165

**Published:** 2022-10-04

**Authors:** Tianhui Jiang, Lorenza Maddalena, Julio Gomez, Federico Carosio, Alberto Fina

**Affiliations:** 1 Department of Applied Science and Technology, Politecnico di Torino, Alessandria Campus, V.le Teresa Michel 5, 15121 Alessandria, Italy; 2 AVANZARE Innovacion Tecnologica S.L., 26370 Navarrete, La Rioja, Spain

**Keywords:** polyelectrolytes, reduced graphite oxide, water dispersion of graphene related materials

## Abstract

The polyelectrolyte (PE)-based water dispersion of graphene-related materials (GRMs) represents an interesting intermediate for the development of advanced materials by sustainable processes. Although the proof of concept has been demonstrated, there is a lack of knowledge for what concerns the effects of parameters typical of PEs such as functionalization, molecular weight, and charge density. In this work, we evaluate the effects of such parameters on the quality and long-term stability of reduced graphite oxide (rGO) dispersion in aqueous media prepared by ultrasound sonication in the presence of different PEs. Four PEs were evaluated: polyacrylic acid (PAA), branched poly(ethylenimine) (BPEI), sodium carboxymethyl cellulose (CMC), and poly(sodium 4-styrenesulfonic acid) (PSS). The prepared dispersions were thoroughly characterized by means of UV-visible spectroscopy, thermogravimetric analysis, dynamic light scattering, and Raman spectroscopy. The highest concentrations of rGO were achieved by BPEI with a molecular weight of 25,000 and 270,000 Da (33 and 26 µg/mL, respectively). For other PEs, the rGO concentration was found to be independent of the molecular weight. The PAA-based dispersions displayed the best through-time stability while yielding homogeneous dispersion with a smaller average size and narrower size distribution.

## 1. Introduction

Due to their exceptional electrical, thermal, and mechanical properties, graphene and graphene-related materials (GRMs) have been intensively investigated and are considered the key material capable of enabling substantial advances in a wide range of applications [1,2,3]. Unfortunately, the strong π–π stacking and van der Waals interactions between graphene sheets naturally prevent an easy and straightforward use of this 2-dimensional (2D) material. Thus, huge research efforts have been focused on the stabilization of GRMs in different liquid media in order to obtain stable dispersions enabling viable graphene processing. However, the limited compatibility and poor stability of the produced suspension still prevent the large-scale exploitation of GRMs in several applications such as functional fluids [4], thin films [5,6], and polymer composites [7,8]. The most commonly employed solvents are N-methyl-2-pyrrolidone (NMP) and N,N-dimethylformamide (DMF), which allow for the proper dispersion of graphene by direct exfoliation via ultrasonication [9,10], but the high boiling points and toxicity limit their wide use [11,12]. On the other hand, water is more attractive than organic solvent owing to its non-toxicity, extremely low cost, and ease of removal. Covalent and non-covalent modifications have been widely pursued in the production of stable aqueous dispersions [13,14,15]. The non-covalent method is considered a better choice due to its reduced impact on the GRM structure and, consequently, its properties [16,17]. In recent years, numerous chemicals, involving surfactants and polymers (polyelectrolytes, PEs and biopolymers), have been investigated to produce GRM dispersions in water [15,18,19]. These chemicals generally bind to the graphene surface, minimizing the interfacial energy with water, and thus resulting in a stabilization effect [19,20]. A wide variety of ionic and non-ionic surfactants have been investigated by exploiting different interactions such as surface adsorption, micelle formation, and/or π-π stacking [18,21]. As a general principle, non-ionic surfactants exploit steric interactions to prevent re-aggregation. On the other hand, ionic surfactants can impart effective charges to graphene layers, leading to electrostatic repulsion effects to control the stability of the dispersions [13,21]. Amphiphilic molecules used as ionic surfactants include polyaromatic molecules such as pyrene- or perylene-bearing polar groups [22,23,24]. Polyaromatic structures are indeed known to strongly interact with graphene via π–π stacking, which provides sufficiently strong non-covalent bonding without compromising graphene properties. Similarly, non-covalent bonding may also occur between hydrophobic polymers and GRMs [16,25,26]. Therefore, PEs in which hydrophobic and hydrophilic parts coexist might also be considered for the preparation of GRM water suspensions [16,27]. For example, poly(sodium 4-styrenesulfonic acid) (PSS) benzene rings offer the possibility of π-π stacking with graphene sheets, while the sulfophenyl groups allow for hydrophilic interactions [28,29]. Up to now, PEs have been widely investigated in empirical tests to assist the dispersion of GRMs by combining them with other, different, auxiliary means, including the addition of organic solvents/surfactants [6,30], the exploit of the in-situ reduction of graphene oxide to form covalent or non-covalent functionalization [31,32], and the chemical modification of polyelectrolytes, such as pyrene-terminated polyacrylic acid (py-PAA) [33,34]. In order to achieve aqueous dispersions of rGO, the combined use of PEs and other stabilizers was recently investigated: for example, hexadecyltrimethylammonium (CTAB)-exfoliated graphite followed by branched poly(ethylenimine) (BPEI) modification [30] or polyacrylic acid (PAA) coupled with poly(ethylene glycol)-block-poly(propylene glycol)-block-poly(ethylene glycol) (PEG-PPG-PEG) was employed [6]. Another approach is to exploit the strong ionic and/or hydrogen bond interaction occurring between GO and PEs by performing GO reduction in the presence of the PE of choice [35,36,37]. Interestingly, few PEs can yield synergistic reduction effects while exerting their main stabilizing function. For instance, poly(diallyldimethylammonium chloride) (PDAC) not only induces the reduction process but also improves the stability of the resulting PDAC-modified rGO (PDAC-rGO) dispersions due to electrostatic repulsion [38,39,40]. Although the literature background has clearly highlighted the potentialities of different PEs in producing rGO dispersions, there are only few research works focused on the direct stabilization of graphene sheets with assistance only of PEs. Moreover, most published works have studied a single PE or a set of stabilizer molecules with few PEs included. Viinikanoja [41] compared some ionic molecules including six ionic surfactants and two PEs, in which the best colloidal stability, based on the zeta potential of graphene flakes, was obtained with PSS and BPEI. Lu et al. [42] studied the stability of aqueous suspensions of exfoliated graphite nanoplatelets (GNP) with few polyelectrolytes, namely, PDAC, PSS, BPEI or PAA, showing that approximately half of the initial GNP was retained after 48 h in PSS and BPEI dispersions. The production of water-based PE-stabilized GRM dispersions is of scientific interest as they can be used for the solution-based processing of graphene, layer-by-layer fabrication of graphene-based films and membranes [37,43], as well as the complexation to another oppositely charged PE to form GRM-reinforced polyelectrolyte complexes (G-PECs) [44]. Therefore, a comprehensive understanding of the interaction mechanism of PE on graphene sheets in water is required and essential to allow sustainable applications of graphene-based materials. In this study, we systematically investigated the effectiveness of PEs bearing different chemical structures for the production of stable rGO dispersions in water. The interactions between rGO particles and weak or strong polyelectrolytes have been studied by combining a complementary set of characterization techniques considering different PE molecular weights. Moreover, the interactions between weakly charged PEs and rGO were further studied at different charge densities as a function of the dispersion pH.

## 2. Materials and Methods

### 2.1. Materials

PAA (35 wt% in water, *M*_w_~100,000 Da, *M*_w_~250,000 Da), PSS (30 wt%, *M*_w_~70,000 Da, *M*_w_~250,000 Da), CMC (*M*_w_~90,000 Da substitution degree 0.7, *M*_w_~250,000 Da, substitution degree 1.2), and BPEI (*M*_w_~25,000 Da, *M*_w_~270,000 Da) were purchased from Merck (Darmstadt, Germany) and used as received. Pristine rGO was obtained by the oxidation of graphite, ultra-sonication, and then thermal reduction of GO according to a previously reported method [45]. The surface area of BET is 196 m^2^/g, the oxygen percentage, atomic, by XPS is 2% [46]. All solutions and dispersions were prepared using deionized water (Direct-Q^®^ 3 UV Millipore System, Milano, Italy).

### 2.2. Preparation of PE/rGO Dispersions and Films

**Figure 1:**
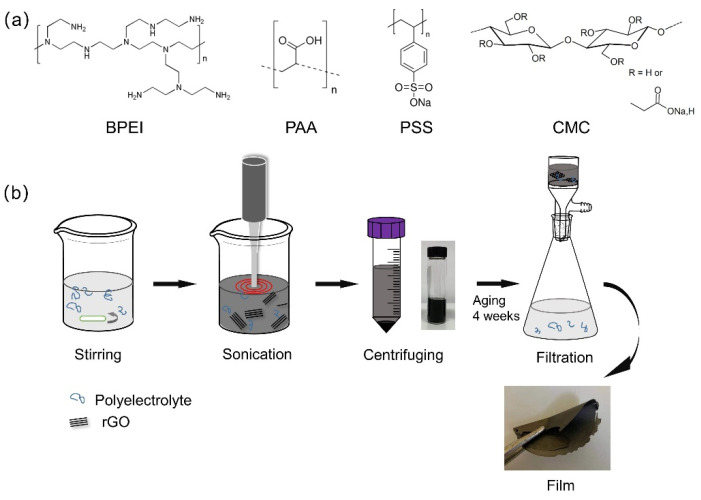
Chemical structure of selected PEs (**a**); illustration of the fabrication process (**b**).

The selected PEs were used for dispersing rGO in aqueous solution following the preparation process schematized in Figure 1. PE 0.1 wt% water solutions were prepared under magnetic stirring at room temperature. After stirring for 15 min, pristine rGO (25 mg) was added to the PE solution (100 mL) and sonicated for 30 min, using a probe sonicator VC-505 (Sonics & Materials, Newtown, CT, USA) with 15 s ON/15 s OFF pulses cycles, 20 kHz frequency, and 30% amplitude. The measured amount of energy per unit volume in 30 min sonication was ~344 J/mL. In this step, the standard sonic probe with threaded end and replaceable tip (model 630-0210, Sonics & Materials, Newtown, CT, USA) was immersed into solution, and the distance from the top of probe to the bottom of the beaker was set to 10 mm. The so-obtained suspension was allowed to settle overnight. Then, the PE/rGO dispersions were centrifuged for 15 min twice at 3300 rpm (Eppendorf centrifuge 5702, Milano, Italy), and the black or grey supernatant was collected. Films of PE/rGO were obtained by gravimetric filtration of the supernatants using polycarbonate membrane with a 0.2 μm pore size and then dried in an oven at 60 °C for 24 h. The employed PE solutions are herein referred to with codes containing the polyelectrolyte name followed by the mass in kDa. For example, the PAA with *M*_w_ 100,000 Da is coded as PAA100. Similarly, the dispersion stabilized with PAA100 is referred to as PAA100/rGO, and the same coding format was applied to the other dispersions.

### 2.3. Characterization

The contact angle was qualitatively measured by dropping the PE solution on rGO film. rGO (30 mg) was loaded in 100 mL deionized water (in a 250 mL beaker) and subsequently sonicated for 30 min with 15 s ON/15 s OFF pulses cycles. At least 200 mL supernatant solution was filtered with a polycarbonate membrane with 0.1 um pore size. Water or 0.1 wt% PE solution with 10 μL was placed on the resulting films using a microsyringe, and the images of the liquid droplet were obtained instantaneously using a camera. The contact angle of the solid and liquid interface was obtained by measuring the angle between the tangent lines to the droplets and baseline. Five measurements for each solution were taken at different areas of the rGO film.

UV-vis spectra were recorded in a wavelength range of 200–700 nm (Shimadzu 2600 UV-vis Spectrophotometer, Kyoto, Japan), using a quartz cuvette (Exacta-Optech^®^, Modena, Italy) with a path length of 10 mm and a volume of 3.5 mL. The characterization of the stability of the PE/rGO dispersions was based on the fraction of rGO remaining for every dispersion after aging, which can be calculated from the ratio of the absorption intensity A_G_/A_G,i_ of the rGO dispersed after aging (A_G_) to that before aging (A_G,i_).

Additionally, the dispersed concentration of rGO was obtained according to UV-vis spectroscopy and the Beer-Lambert law (*A* = *αcL* where *α* is the absorption coefficient, *c* is the concentration, and *L* is the path length). The first measurement of *c* used to determine *α* was obtained by filtration, drying, and weight measurement on a high-sensitivity and resolution balance. The detailed procedure for calculating the concentration and different *α* values (in Figure S1) of each sample is provided in Section S1 in supporting information (SI). Four samples were analyzed for each dispersion to provide average values and experimental deviations. Dynamic light scattering (DLS) measurements were evaluated by a Malvern Zetasizer Nano ZS90 (Malvern Instruments, Malvern, UK) at 25 °C with a refractive index of solvent (water) 1.33. Every sample was tested three times, and the results were averaged.

Raman spectra were assessed on a Renishaw Invia Raman microscope system (Renishaw, Wotton-under-Edge, UK) equipped with a 532 nm laser. A 20× objective lens was used for measurements. Raman spectra were collected at 3 random points for each sample, and the average spectrum was used to characterize the filtered sample. Thermogravimetric analysis (TGA) was performed with a Q500 thermobalance (TA Instruments, Newcastle USA) under nitrogen flux. The samples were held isothermally at 100 °C for 30 min to remove weakly adsorbed water, followed by a heating rate of 10 °C/min from 100 °C to 800 °C.

The rGO weight fraction (%) was determined by Equation (1).

(1) $$w_{G}=\frac{M-M_{PE}}{M_{G}-M_{PE}}\times 100\%$$

where *w_G_* is the weight fraction of rGO in the PE/rGO composite on the filter membrane; *M, M_PE_*, and *M_G_* represent the residual weight of PE/rGO, pure PE, and rGO, respectively.

## 3. Results and Discussion

### 3.1. Dispersion and Stability of rGO in Polyelectrolyte Solutions

#### 3.1.1. Visual Observation

PE/rGO dispersions were prepared by directly adding rGO in PE solutions and then sonicating under relatively mild conditions. It is interesting to note that within a few seconds after rGO came into contact with the liquid surface, the rGO powder rapidly produced a film at the water/air interface for PAA and BPEI solutions but not in water, PSS or CMC solutions; see video S1. In addition, it was clearly observed that a limited amount of rGO was included in the PAA and BPEI solutions after one night of storage. This was more evident for PAA solutions, see Figure S2. The observed, practical behavior can be ascribed to a change in the surface tension of the PE-containing water-based solution. It is indeed well-known from the literature that the type and concentration of the PE might affect this value to different degrees. The values of the surface tension of the PE aqueous solutions, extrapolated from the literature background, are summarized in Table 1.

When the PE concentration was close to 0.1 wt% (the concentration employed in this work), the PSS, CMC, and BPEI solutions showed surface tensions similar to that of water (≈72 mN m^−1^), which was expected at such low concentrations. Conversely, PAA was reported to have a lower surface tension, even at 0.1 wt%. This latter behavior is explained by the fact that PAA macromolecules consist of non-polar hydrocarbon backbones that tend to orient toward the surface of the solution to minimize the surface tension [55]. When rGO comes into contact with the surface of the aqueous PE solution, the interplay of three interfacial tensions determines the wettability of the rGO, expressed as cosθ, following Young’s equation [56]:

(2) $$r_{sg}=r_{sl}+r_{lg}\cos\theta$$

where *γ_sg_*, *γ_sl_*_,_ and *γ_lg_* are the solid-air, solid-liquid, and the liquid-air interfacial energy, respectively. The solid surface free energy can be determined by several theoretical models or through direct contact angle measurements [57]. The surface tension of rGO was estimated to be ~47 mN/m by Neumann’s method [58]. On the basis of the state equation, the contact angle is given as Equation (3).

(3) $$\cos\theta =-1+2\sqrt{\frac{\gamma_{sg}}{\gamma_{lg}}}e^{-\beta {\left(\gamma_{sg}-\gamma_{lg}\right)}^{2}}$$

where *β* is the constant coefficient related to a specific solid surface. *γ_sg_* and *γ_lg_* represent solid and liquid surface free energy, respectively [59]. By combining Equation (3) and the values reported in Table 1, it is immediately apparent that a lower *γ_lg_* would lead to a smaller contact angle.

**Figure 2:**
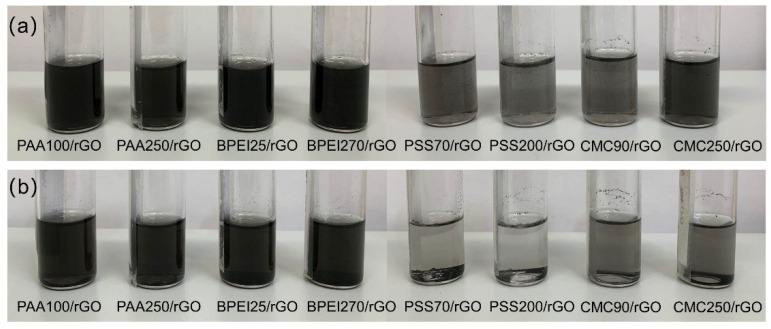
Photographs of rGO dispersed in different polyelectrolyte solutions before (**a**) and after four weeks of aging under static conditions (**b**).

Therefore, PAA had a smaller contact angle when compared with water or other PE solutions in our study. This was confirmed by practically evaluating the wettability of rGO films as shown in Figure S3. Indeed, the PAA solutions showed better wettability, or smaller contact angles, than other PEs. This could explain the inclusion of rGO clusters within the PAA solution without the need for external energy inputs. It is worth mentioning this fast rGO inclusion in the liquid was exclusively formed in PAA solutions at low pH values (namely, within pH 2 and pristine pH 3.4), which suggests this being related to the low ionization degree of PAA at such pH conditions. Indeed, as reported by Brittany et al. [47], the surface tension of PAA solutions dramatically increased in the pH 4–7 range pH as the PAA was mostly ionized. Thus, rGO was not expected to be incorporated into the liquid rapidly in the PAA solution with a pH higher than 5, which was consistent with the observation (video S2). On the other hand, BPEI also revealed ease of rGO inclusion in the liquid phase (video S1) despite its high surface tension, which suggests a possible electrostatic effect as a driving interaction [45]. Aiming at the separation of aggregated rGO flakes, rather than their extensive exfoliation, 30-min pulsed sonication was applied in this work, which is significantly shorter than that conventionally employed in exfoliation procedures of graphite, where sonication times range from tens to hundreds of hours [60,61]. The appearance of the dispersions obtained immediately after sonication can represent a first qualitative evaluation of the quality of the rGO dispersion. From an overall point of view, the darker the supernatant, the better the dispersion, with a higher concentration of suspended rGO. However, the appearance of the just-prepared dispersion is not representative of its long-term stability, which should be evaluated in time. Figure 2 shows the images of different PE-stabilized rGO dispersions before and after four-week storage under static conditions. The darkest rGO dispersions were obtained in PAA-based (both PAA100/rGO and PAA250/rGO) and BPEI-based dispersions (both BPEI25/rGO and BPEI270/rGO). Light grey supernatants were obtained for PSS-based (PSS70/rGO and PSS200/rGO) and CMC-based dispersions (CMC90/rGO and CMC250/rGO). This suggests that a higher content of rGO was dispersed in the PAA-based and BPEI-based dispersions after preparation. After storage for four weeks, rGO in PSS-based dispersions was almost completely precipitated (Figure 2b). CMC-based dispersions also showed conspicuous sedimentation. On the other hand, the PAA-based and BPEI-based dispersions remained qualitatively equivalent, suggesting good stability over four weeks.

#### 3.1.2. UV-vis Characterization

**Figure 3:**
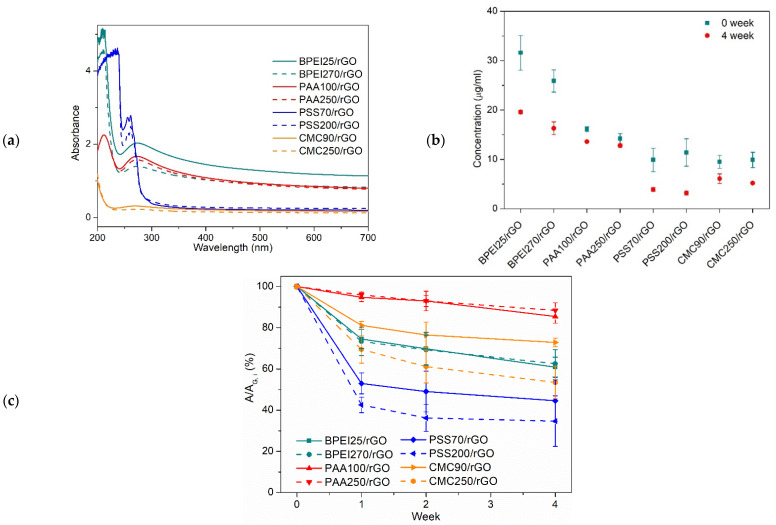
UV-vis spectra of PE/rGO dispersions (**a**); concentration of rGO in PE/rGO dispersions (**b**); the amount of remaining rGO in dispersions after 0 to 4 weeks, expressed as the percentage of the original absorption (original concentration), A/A_G,i_ (**c**).

UV-vis spectroscopy was used to investigate and quantify the rGO concentrations in the prepared PE solutions and their stability through time. Figure 3a displays the UV-vis spectra of PE/rGO dispersions. Once the signal of the employed PEs was evaluated (see Section S3 in SI), the presence of rGO was related to the appearance of an additional signal at a wavelength of ~270 nm, ascribed to the sp^2^-conjugated graphene network [62]. Moreover, the addition of rGO increased the overall optical density of the dispersion within 200–700 nm; as a consequence, the signal baseline shifted from the zero of neat PE solutions to variable values for PE/rGO dispersions. As the increased absorbance intensity is dominated by the concentration of graphene sheets [8,19], the shift of the baseline can be related to the difference in the amount of rGO dispersed in each PE. The selected different molecular weights had little effects on the collected spectra of most samples as shown, for example, by comparing PAA100/rGO and PAA250/rGO. On the other hand, the absorption intensity of BPEI25/rGO was significantly higher than that of BPEI270/rGO, suggesting a better dispersion concentration of rGO for the former. The calculated concentrations of rGO for each prepared dispersion before (filled square) and after (filled round) four weeks are reported in Figure 3b. The initial dispersed concentration of rGO follows the trend: CMC90/rGO ≈ CMC250/rGO < PSS70/rGO < PSS200/rGO < PAA250/rGO < PAA100/rGO < BPEI270/rGO < BPEI25/rGO. This is in agreement with the qualitative considerations based on rGO wettability described at the beginning. After four weeks, the concentration remained almost unchanged only for PAA-based dispersion, whereas decreases were observed for BPEI- and PSS-based dispersions (Figure 3b). In addition, the precipitation of large and unstable aggregates through the aging period led to a considerably decreased experimental error in the measured concentration for all prepared dispersions.

In addition, the UV-vis spectra were also employed to follow the stability of rGO dispersions as a function of time (i.e., one, two, and four weeks). The residual rGO fraction, expressed as a% of the initial concentration, is plotted in Figure 3c. Most PEs clearly showed that relatively large fractions of rGO had sedimented during the first week, after which limited reductions are observed. Interestingly, PAA-based dispersions yielded very low sediment fraction over the whole period (four weeks). In addition, it was also possible to observe the effect of different molecular weights. Indeed, for both CMC and PSS, lower-molecular-weight PEs led to relatively higher stability in time. On the other hand, the gaps between PAA100/rGO and PAA250/rGO and between BPEI25/rGO and BPEI270/rGO were narrow, thus suggesting a limited effect of the molecular weight on the stability over time for these PEs. Interestingly, although the original absorbance of BPEI25/rGO was slightly higher than that of BPEI270/rGO (in Figure 3a), their absorbances decreased in time by equal proportions. In addition, although the BPEI-based samples showed quite drastic reduction in concentration during aging, the initial amount of dispersed rGO was so high that even after four weeks, these dispersions still displayed the highest concentrations among all PEs. Combined with the results in Figure 3b,c, it was concluded that, among the different PEs addressed, BPEI-based dispersions showed the highest concentration of rGO, and the PAA-based dispersions exhibited the best stability over four weeks.

### 3.2. Quantification of PE Adsorbed to rGO

To further investigate the mechanism of stabilization of rGO dispersions, it is of importance to determine the amount of PE adsorbed on rGO in dispersion (PE: rGO) over time. For this aim, TGA was conducted for pristine rGO, Pes, and PE/rGO films obtained after filtration, as shown in Section S4 of SI (Figure S5). Pristine rGO showed a mass loss of 4.9% upon heating to 800 °C under nitrogen, indicating a relatively small amount of oxygen-containing groups [63]. The selected PEs showed a different weight loss behavior. Indeed, BPEI starts decomposing at 250 °C, and above 650 °C, it is completely volatilized [44]. PAA yielded two decomposition steps, the first assigned to the thermal decomposition of the carboxylic acid groups at around 280 °C and the second reflecting the scission of the polymer backbone [64], eventually leading to a~15% residue. CMC exhibited a first main mass loss in a temperature range of 260 to 300 °C, assigned to the loss of CO_2_ from decarboxylation coupled to the pyrolytic decomposition of the main chain followed by further mass loss ascribed to aromatization, the final residues accounting for 35% (CMC250) and 27% (CMC90) [65,66]. PSS showed a more complex and multi-step decomposition path with an onset temperature of mass loss at approx. 400 °C and a final residual weight around 35% [67]. The TGA curve patterns of PE/rGO composite films were similar to their corresponding PEs, suggesting that the decomposition mechanism was not altered by the presence of rGO. The final residues fell within the values obtained for the neat film constituents, thus allowing for the evaluation of the relative PE/rGO amounts. Table 2 reports the weight residual of PE/rGO within the 100–800 °C temperature range, the weight percentage of rGO *w_G_*, and the corresponding fraction of PE in the PE/rGO composite (calculated as 1*−w_G_*). rGO accounted for more than 60 wt% in all cases, with some differences between the different PEs. The (1*−w_G_*) values of PAA and BPEI were similar, whereas CMC had the highest ratio in the PE/rGO composite and PSS the lowest value. Combined with previously measured concentrations, these results suggest that the fraction of PEs attached on the rGO basal plane was relatively low compared to the total in solution, leaving a significant excess of PEs solubilized in water.

### 3.3. Size and Defectiveness of Suspended rGO

**Figure 4:**
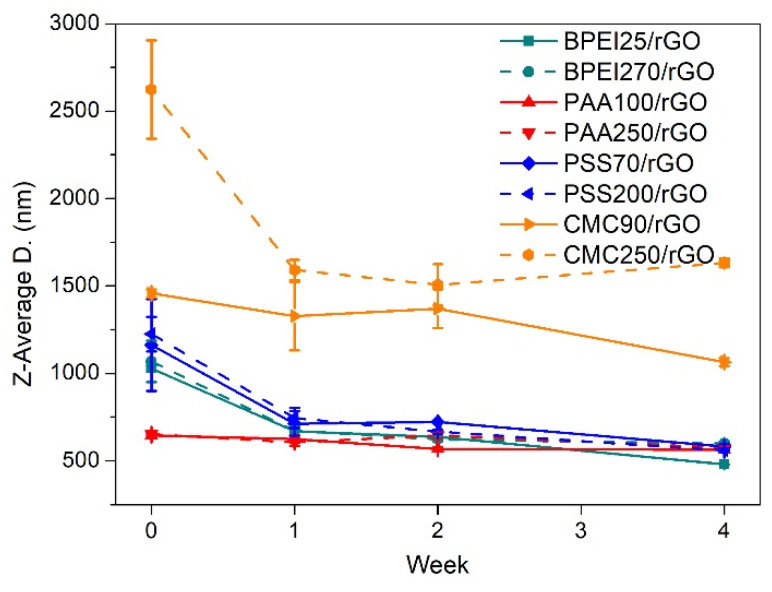
Average size of suspended rGO with different aging times.

To investigate the evolution of suspended particle size vs. time, DLS measurements were carried out. DLS was used for measuring the size distribution of aqueous dispersion of GRMs in previous studies [41,68]; however, it should be noted that this could only be considered a semi quantitative analysis because of the complex shapes of GRMs and is therefore taken as a purely comparative value between different dispersions. The Z-average is the harmonic intensity average particle diameter, a measure of the average hydrodynamic diameter, and corresponds to the radius of an equivalent hard sphere diffusing as the same rate as the particle under observation [69,70]. Figure 4 displays the Z-average for the different PE/rGO dispersions during the four weeks. PAA100/rGO and PAA250/rGO yielded the smallest initial Z-average, almost half that of BPEI/rGO and PSS/rGO. The initial measurements for CMC90/rGO and CMC250/rGO were much higher than those of other samples. The average size of the suspended rGO generally decreased with time (Figure 4), suggesting the precipitation of larger sized rGO. PSS-based and BPEI-based dispersions showed a significant Z-average size decrease within the first week, suggesting that most of the rGO precipitation occurred in this time frame. CMC exhibited a downward trend in the overall period but a much higher Z-average than that in other groups. PAA100/rGO and PAA250/rGO showed the most stable Z-average during the whole four weeks (PAA100/rGO changed from 644 ± 5 nm to 563 ± 4 nm, PAA250/rGO varied from 652 ± 3 nm to 575 ± 8 nm), thus confirming a better long-term stability of the prepared dispersion. Such a result is consistent with the UV–vis measurements reported in Figure 3b. In addition, the light scatter analysis provides insight into the reasons behind the better stability obtained with PAA, which appears to be directly related to the reduced particle size dispersion with respect to other PE systems. This suggests that PAA, compared to other PEs under study, is more efficient in limiting the aggregation of rGO into larger and poorly suspended clusters. The polydispersity index (PDI) was also evaluated before and after aging (Table S1). PDI is a dimensionless parameter that indicates the width of the detected particle size distribution, which is used to quantify the quality of the dispersion [71]. When PDI < 0.1, the sample is defined as monodisperse, values in the range of 0.1 < PDI < 0.2 indicate narrow particle size distributions, and 0.2 < PDI values < 0.5 are normally obtained for broad particle size distributions [71]. In PAA100/rGO and BPEI25/rGO, the smallest and largest PDI values of 0.19 and 0.62 were obtained, highlighting a strong difference in the particle size distribution. Of all the tested PEs, only PAA100 and PAA250 obtained a PDI close to 0.2, thus suggesting their superior stabilizing ability with respect to the other tested PEs. It is worth noting that the PDI decreased for almost all systems after aging likely due to the sedimentation of the large particle fractions.

**Figure 5:**
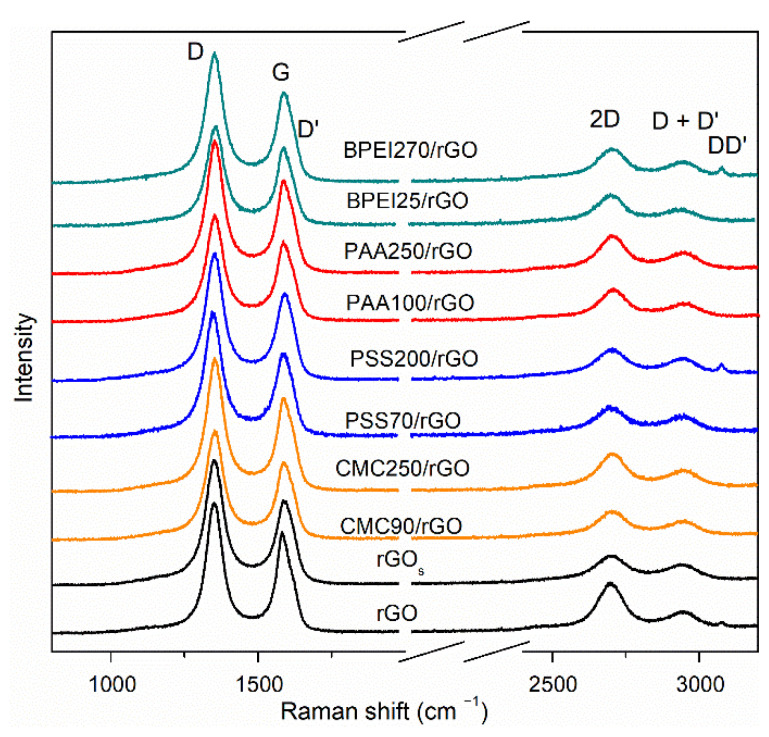
Raman spectra of rGO with PEs. For comparison, the spectra of pristine rGO and rGOs after sonication in water are also provided.

The structural quality of the dispersed rGO was further investigated by Raman spectroscopy, which represents a well-known technique to evaluate defectiveness in GRMs [72]. Figure 5 presents the spectra of the PE/rGO composite film, pristine rGO, as well as the sonicated rGO (rGO_s_). The latter was treated in water with the same process employed for PE-based dispersion in order to evaluate the effects of sonication on the defectiveness of the pristine rGO. As shown in Figure 5, two main sets of signals were observed in pristine rGO, rGO_s_, and other PE/rGO samples. The first-order of signal (1200–1700 cm^−1^) is composed of D, G, and D′ bands. The D band is related to the breathing modes of six-atom rings, and it requires a defect for activation, while the G band is ascribed to the in-plane stretching vibration mode of sp^2^ carbon atoms [73,74]. In the second-order, the 2D band, D + D′ band, and DD′ band were observed. The 2D band and DD′ band are the overtones of the D band and D + D′ band, respectively [75,76]. It is well established that the relative intensity of the D band to the G band (I_D_/I_G_) and full-width at half maximum of the G band (Γ_G_) can be associated with the disorder of the graphene material network [74]. The I_D_/I_G_ ratio was comparable at 1.41 ± 0.17 and 1.45 ± 0.06 for rGO and rGO_s_, respectively. Meanwhile, the Γ increased in the D band, and a very relevant increase was observed in Γ_G_, indicating the formation of new edges or a slight increase in the defect content due to the sonication treatment [75,77]. After being dispersed within the PEs, the calculated I_D_/I_G_ values were similar to rGO_s_ and fell within the range of 1.40–1.45 except for BPEI25/rGO, which had a lower ratio value of 1.26 ± 0.09. In addition, the slightly lower Γ_G_ and position of the G band suggested a weak reduction effect caused by BPEI, that was previously reported [78,79]. Overall, the Raman results clearly point out a limited effect on the defectiveness of rGO, which is consistent with the non-covalent interaction between PE and rGO.

### 3.4. Interaction between PE and rGO

#### 3.4.1. Charge Density of PAA and BPEI

**Figure 6:**
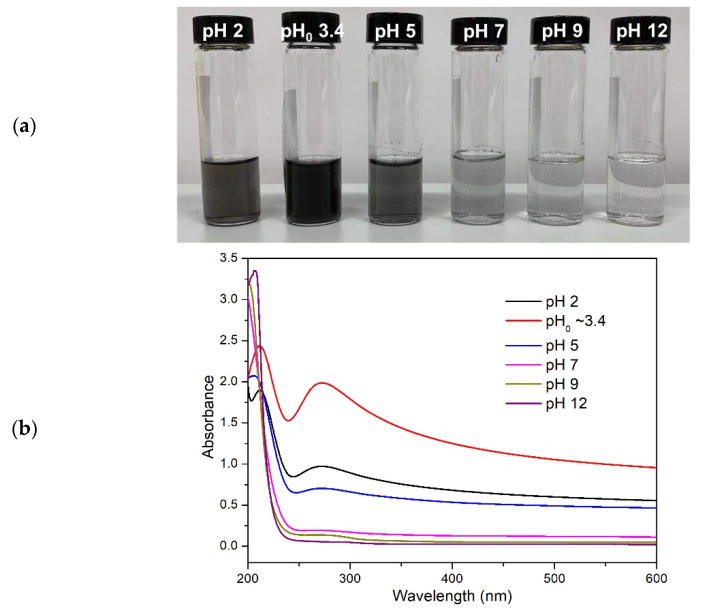
Photographs (**a**) and UV-vis spectra (**b**) of PAA100/rGO with different pH values.

The above results clearly demonstrate the highest concentration of rGO in the BPEI-based dispersion and the remarkable stability of PAA/rGO dispersion. Considering that both BPEI and PAA are weak PEs, their surface charge density is tunable with pH, which makes it possible to adjust the interaction between PE and rGO by reinforcing or weakening electrostatic interactions. Due to the similar dispersion and stability of PAA100 and PAA250 in the above results, PAA100 was investigated. Conversely, BPEI25 was selected due to the high concentration of rGO obtained. rGO is well known to have weak negative charges in water due the residual functional groups [80]. As the charge on rGO is weak, the adsorption of PAA to the rGO surface is made possible by attractive hydrophobic interactions occurring between the PAA backbone and rGO. The negatives charges (-COO^−^) on PAA then provide an effective repulsive force to prevent the re-stacking of rGO sheets [45]. The interaction between rGO and PAA as well as the surface tension of the PAA solution is strongly related to the dissociation degree of PAA functional groups that can be modified by changing pH. Aiming at further investigation of the nature of the interaction between PAA and rGO, a series of PAA100/rGO with variable pH was prepared (Figure 6). The unmodified dispersion (pH 3.4) showed the highest intensity of absorbance in UV–vis spectra, corresponding to a higher concentration of rGO, which can be qualitatively appreciated by visual inspection as it yielded the darkest dispersion. According to the Henderson–Hasselbach equation, the degree of ionization α was calculated to be around 0.12 (see Section S6 in Supplementary Materials), which means that the functional groups were predominantly protonated, and a limited fraction of the carboxyl groups was dissociated to –COO–. At pH 2, α was 0.005, which resulted in limited repulsion interaction to counterbalance the van der Waals forces and π–π-driven rGO re-stacking. On the other hand, at pH 5, α strongly increased to 0.84, leading to strong repulsion forces that hindered the close contact between rGO and PAA. When pH reached 7 or above, α was further increased towards the full dissociation of PAA. At this point, it was extremely hard for PAA to adsorb onto graphene. Therefore, the good dispersion performance of rGO in PAA appeared to be the result of an optimum balance of both electrostatic repulsions and hydrophobic interactions.

**Figure 7:**
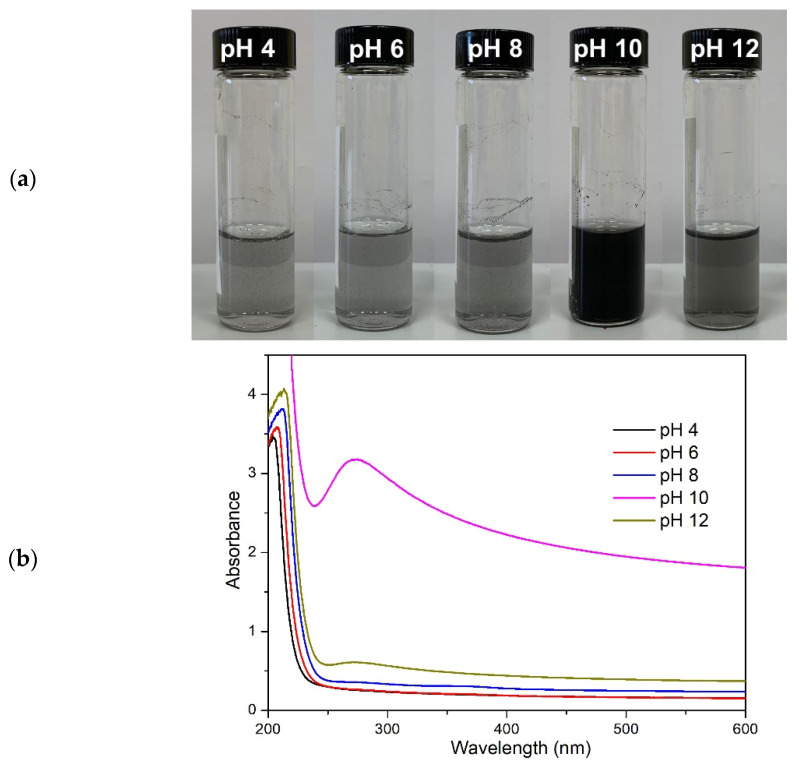
Photographs (**a**) and UV-vis spectra (**b**) of BPEI25/rGO with different pH values.

Conversely, BPEI is a cationic hydrophilic PE bearing primary, secondary, and tertiary amines, and carbon spacers. Based on the positively charged amines, an attractive electrostatic force between BPEI and the rGO surface is expected [45]. Being classified as a weak PE, the charge density of BPEI can also be tuned by the pH. Indeed, its protonation degree increases gradually from 0 at pH 12 to 1 at pH 2, with three pKa values for primary, secondary, and tertiary amines calculated as 4.5, 6.7, and 11.6, respectively [47,81]. In order to evaluate the adsorption behavior of BPEI with respect to rGO, a series of BPEI25 solutions with different pH values, from pH 4 to pH 12, were used to prepare BPEI/rGO dispersions (Figure 7). With decreasing degree of protonation, i.e., by increasing the pH, it was possible to note that the concentration of rGO increased by moving from pH 4 to 10 and then dropped in the 10 to 12 pH region. The uncharged BPEI solution (pH 12) revealed better rGO dispersion performances with respect to that of solutions characterized by an ionization degree α higher than 0.4 (pH ≤ 8). These results are in contrast to our previous hypothesis that BPEI and rGO interactions were mainly electrostatic in nature. However, these results are in agreement with a previous study describing the adsorption of BPEI on uncharged graphite [82]. Indeed, the adsorption process of BPEI onto graphite could be divided into three steps: diffusion toward the surface, adhesion to the surface, and rearrangement [82]. Step two plays an important role in dispersing and stabilizing rGO, and it is influenced by the intramolecular segment-segment repulsion in BPEI. At lower pH, BPEI is highly protonated and shows a strong repulsive interaction between charged segments. Even if positive to negative charge compensation occurs at the surface of rGO, the interaction is limited based on the huge difference in charge density between rGO and BPEI. Thus, the adsorption of a high number of highly protonated BPEI chains is limited due to charge repulsion effects.

With decreasing charge density (higher pH), the repulsive force is reduced; consequently, the adsorbed amount increases until it reaches a maximum with uncharged molecules. However, as demonstrated in Figure 7a, the best dispersion was obtained at pH 10, not at pH12. This can be easily explained by considering that, after adsorption on rGO, electrostatic repulsion is mandatory in order to achieve a stable dispersion. Thus, obtaining a suitable degree of protonation represents a critical condition for dispersing and stabilizing rGO as it limits BPEI segment-segment repulsion while allowing for optimal attractive van der Waals interactions between rGO and BPEI.

#### 3.4.2. CMC and PSS

In principle, similar interactions (such as PAA to rGO) could also occur in rGO dispersions containing other negatively charged PEs, such as PSS and CMC. CMC consists of β-linked glucopyranose units with varying levels of carboxymethyl (–OCH2COO−) substitution [83]. The degree of solubility (colloidal dispersion or full solubility in solution) is accepted to be function of the degree of substitution (DS) as the carboxymethyl groups are deemed responsible for solubility as cellulose is insoluble in water at room temperature and mild pH [84]. It was reported that CMC with lower DS preferentially adsorbs on graphite due to attractive hydrophobic interactions [85]. This was also verified in this work, where, although the two CMC grades used differ in terms of both molar mass and DS, a greater uptake of CMC on rGO was measured for CMC90 with DS value 0.7 with respect to CMC250 with DS 1.2.

On the other hand, PSS is an amphiphilic polymer in which the hydrophobic benzene ring may provide π–π interactions with the aromatic structure of rGO sheets, while the sulfonate functional groups might, in principle, confer stability to the rGO dispersion by charge repulsion [36,86]. However, the stability of rGO in PSS was limited in this work, leading to about 60% reduction in the concentration of rGO in dispersions after four weeks. This could be explained by considering that, although the hydrophobicity of PSS is much higher than PAA due to the hydrophobic backbone and side chain groups, the steric hindrance exerted by the PSS functional group and the high charge density prevent the adsorption of PSS chains on the surface of rGO.

## 4. Conclusions

Non-covalently functionalized aqueous dispersions of rGO were produced by direct sonication in polyelectrolyte solutions, and the properties of the dispersions were demonstrated to depend on the type of polyelectrolyte used. The dispersions were prepared using polyacrylic acid, branched poly(ethylenimine), sodium carboxymethyl cellulose, and poly(sodium 4-styrenesulfonic acid). The effects of the polyelectrolyte type, molar mass, and charge density on the quality and stability of the dispersion were evaluated. Unlike high-energy exfoliated single or few-layer graphene sheets, this work emphasizes the ability of polyelectrolyte molecules to stabilize reduced graphite oxide under mild ultrasonic conditions, implying a larger lateral size, more layers of graphene layers, and thus more difficulties in maintaining dispersion stability. Moreover, the rarely mentioned relationship between the surface tension of polyelectrolyte solutions and their graphene dispersion ability is discussed, while the surface tension theory is usually applied in organic solvent dispersion systems.

According to the results, the dispersion performance of anionic polyelectrolytes is strongly correlated with the solution surface tension. Polyacrylic acid-based dispersions characterized by low surface tension displayed the lowest average particle size, a narrow size distribution, as well as negligible change in the rGO concentration after four weeks. Conversely, sodium carboxymethyl cellulose and poly(sodium 4-styrenesulfonic acid) showed limited dispersion capability, achieving the lowest rGO concentration. On the other hand, the dispersion effectiveness of cationic polyelectrolytes was not influenced by the starting PE solution surface tension. Indeed, rGO dispersions containing branched poly(ethylenimine) exhibited the highest concentration of dispersed rGO compared with other PEs after four weeks of aging, while the concentration was inversely proportional to the molar mass. For weak polyelectrolytes, the possibility to control charge density through pH has been evaluated as well. When employed at the boundaries of high and low charge densities (i.e., very high or very low protonation and deprotonation degrees), both weak polyelectrolytes under study obtained poor rGO dispersion. Conversely, the optimal dispersion conditions have been found to be related to mild charge densities, allowing for the favorable interplay of electrostatic repulsion/attraction and hydrophobic interactions between the polyelectrolytes and rGO. The results presented here open up the further development of polyelectrolyte/rGO dispersions in water-based assembly processes exploiting electrostatic interactions for the production of membranes and functional coatings.

## Figures and Tables

**Table 1:** Surface tension of PE solutions reported in the literature.

	Temperature (°C)	Concentration (w/v%)	Surface Tension (mN/m)	Method
PAA	25	0.07	~59	Du Nouy ring method (Brittany et al. [47])
	25	0.1	~63	Du Nouy ring method (Elmira et al. [48])
BPEI	25	0.1	~71	Du Nouy ring method (Peter et al. [49])
	25	0.1	~72	Du Nouy ring method (Ismael et al. [50])
CMC	20/25	0.1	~72	Wihelmy method/axisymmetric drop shape analysis method (Samuel et al. [51])
	25	0.5	~75 ~71 ~71	Du Nouy ring method, Harkins-Brown (HB) drop weight method, Lee-Chan-Pogaku (LCP) drop weight method (Boon et al. [52])
		0.25	~69	Du Nouy ring method (Weber et al. [53])
PSS	25	0.1	~72.5	Wihelmy method (Tsuneo et al. [54])

**Table 2:** Weight residual of PE/rGO after TG and the weight percentages of rGO and PE in PE/rGO films.

Sample	Weight Residual (%)	rGO Weight Percentage *w*_G_ (%)	PE Weight Percentage (1−*w*_G_) (%)
BPEI25/rGO	76.1	80.1	19.9
BPEI270/rGO	75.4	79.3	20.7
PAA100/rGO	76.3	76.4	23.6
PAA250/rGO	76.4	77.1	22.9
PSS70/rGO	86.7	86.0	14.0
PSS200/rGO	84.6	82.4	17.6
CMC90/rGO	70.2	63.5	36.5
CMC250/rGO	75.6	67.3	32.7

## Data Availability

The data presented in this study are available on request from the corresponding author.

## Supplementary Materials

The following supporting information can be downloaded at https://www.mdpi.com/article/10.3390/polym14194165/s1, Figure S1: Absorption coefficient (α) of RGO dispersions with different polyelectrolytes; Figure S2: rGO inclusion into PE solutions over one night; Figure S3: The liquid contact angle images of water, BPEI25, PAA100, PSS70, and CMC90 droplets on the surface of rGO with time; Figure S4: UV–vis spectra of PEs with 0.1 wt%, inset: absorption spectra for BPEI25, BPEI270, PSS70, and PSS200 with 0.01 wt%; Figure S5: The TGA curves of PE/rGO after aging for four weeks; Table S1: The PDI values of the PE/rGO samples before and after aging for four weeks. Video S1: rGO inclusion in liquid with different PEs and water; Video S2: rGO inclusion in the PAA solutions at different pH values.
