# Supplementary material for: Polyelectrolytes Enabled Reduced Graphite Oxide Water Dispersions: Effects of the Structure, Molecular Weight, and Charge Density
Source: Polymers (Basel). 2022 Oct 4;14(19):4165. doi: 10.3390/polym14194165 (PMC9573485; doi:10.3390/polym14194165)
Supplement: Supplementary file 1 [file polymers-14-04165-s001.zip › polymers-1832413-supplementary.pdf]

1. Procedure for calculating concentration of exfoliated rGO

The known volume of suspension was filtered through a pre-weighted polycarbonate filter membrane with pore size 0.2 µm, then the deposited mass was measured after drying the membrane for 24h at 60 ºC. Weight fraction of rGO on filter membrane was determined by TGA result. Thus, the concentration of rGO is calculated from equation (1):

$$c = \left((m_1 - m_0) * w_G\right)/V \qquad (1)$$

Where m_1 and m_0 are the mass of filter membrane combined with deposited material, and the mass of pristine filter membrane respectively, V is the volume of dispersion for filtration, w_G is the mass proportion of RGO in deposited material.

UV-vis spectroscopy can be used with only a few millilitres of consumption to quantify the concentration of dispersed graphene flakes based on Beer Lambert Law [1, 2]. According to the Beer Lambert law, the absorption coefficient α was determined by the calculated c and absorption intensity A ($A = \alpha c L$). The coefficients for each samples are reported in Figure S 1. The coefficient α varied among different PE/rGO samples, but for the same type of PE with different molecular weight, the deviation of α was small. Taking corresponding α and A for each dispersion, the concentration after 4 weeks was obtained.

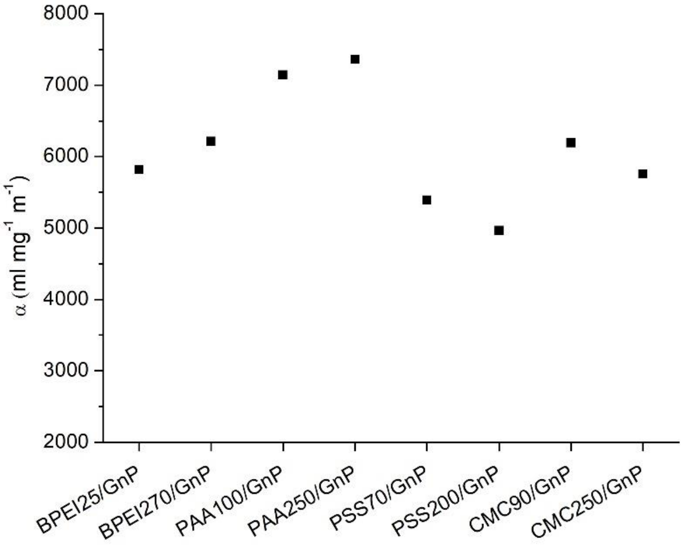

Figure S 1. Absorption coefficient (α) of RGO dispersions with different polyelectrolytes.

2. Images of rGO on PE solutions

Figure S 2 displays the condition of rGO entering into PE liquid phase without sonication over storage one night. It is easy to tell that more inclusion of rGO occurs in PAA-based solutions, subsequently, in the BPEI-based solutions. The PSS and CMC-based solutions show clear and transparent appearance, with hardly found rGO included.

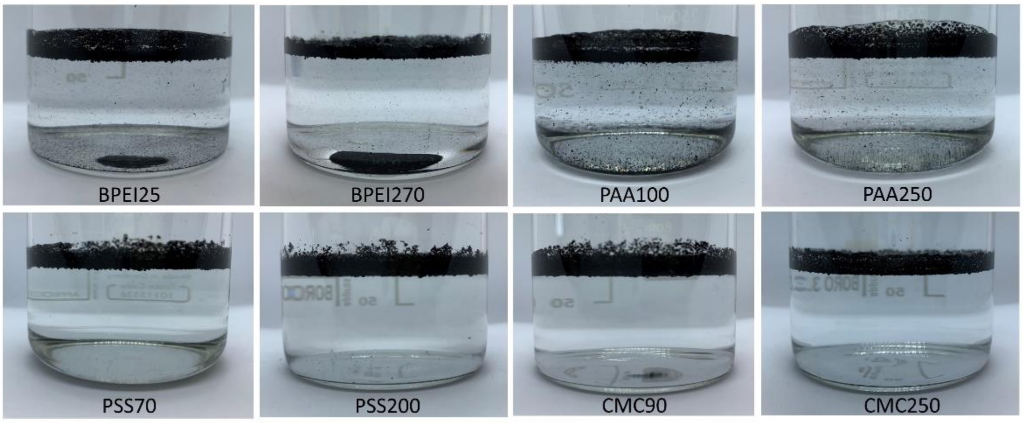

Figure S 2. rGO inclusion into PE solutions over 1 night

Figure S 3 shows the variation of contact angle of PE droplet and rGO film with time. The contact angles of different PE solutions and water are similar within the first few seconds. Deionized water, BPEI25, PSS70 and CMC90 all formed spherical droplets with contact angles higher than 90 °. These droplets kept relatively stable angles on rGO in the first 4 min. However, PAA yielded smaller contact angles from 1 min, corresponding to a fast decrease in droplet height. s. After 10 min, all samples exhibit smaller contact angle than the corresponding initial shape, in which BPEI25, PAA100 and PSS70 have higher variation. This is related to the partial absorption of the solution. From the performed experiments it seems that PAA has a better wettability to rGO when compared with water and other PEs

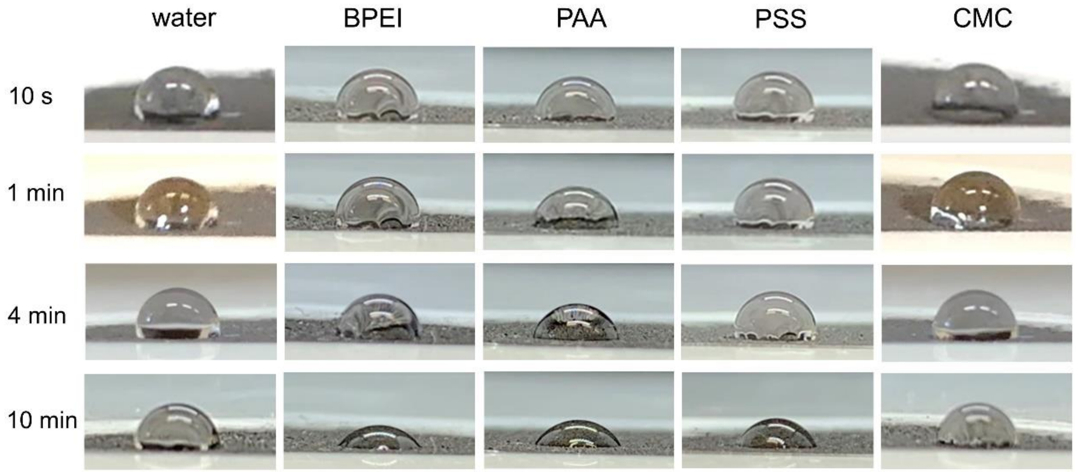

Figure S 3. The liquid contact angel images of water, BPEI25, PAA100, PSS70 and CMC90 droplets on the surface of rGO with time

3. UV-vis of PEs

In Figure S 4, the UV-vis result of PE solutions (0.1 wt% PE) are presented. The spectra of same type of PE with different average molecular weight (Mw) were quite similar. PAA displays an absorbance peak at 212 nm. PSS shows two characteristic shoulder peaks at 256 nm and 261 nm, partially overlapped to the main absorbance, which appears heavily saturated at this concentration. To resolve the position of the PSS main absorbance peak, a 0.01 wt% PSS solution was also analyzed (inset), exhibiting a peak at 222 nm. As for BPEI, a strong absorption is found below 250 nm, which saturates at 0.1 wt% concentration. Even at lower concentration (inset), the peak is not visible, which suggest this to occur at a wavelength lower than 200 nm. Finally, CMC only displays a weak absorbance below 225 nm.

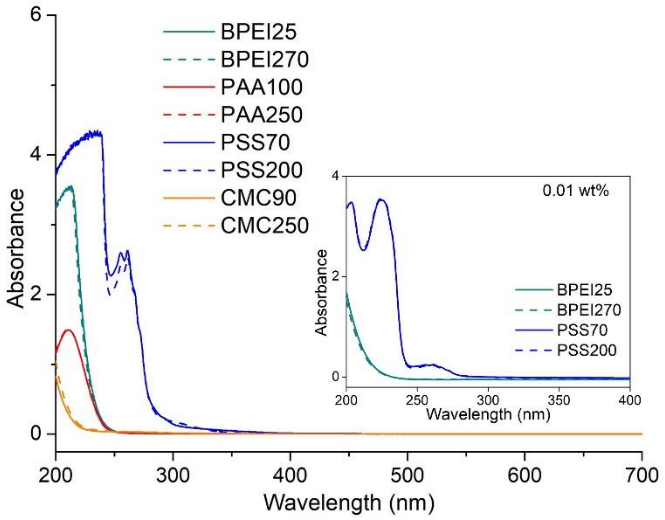

Figure S 4. UV-vis spectra of PEs with 0.1 wt%, inset: absorption spectra for BPEI25, BPEI270, PSS70 and PSS200 with 0.01wt%.

4. TGA test

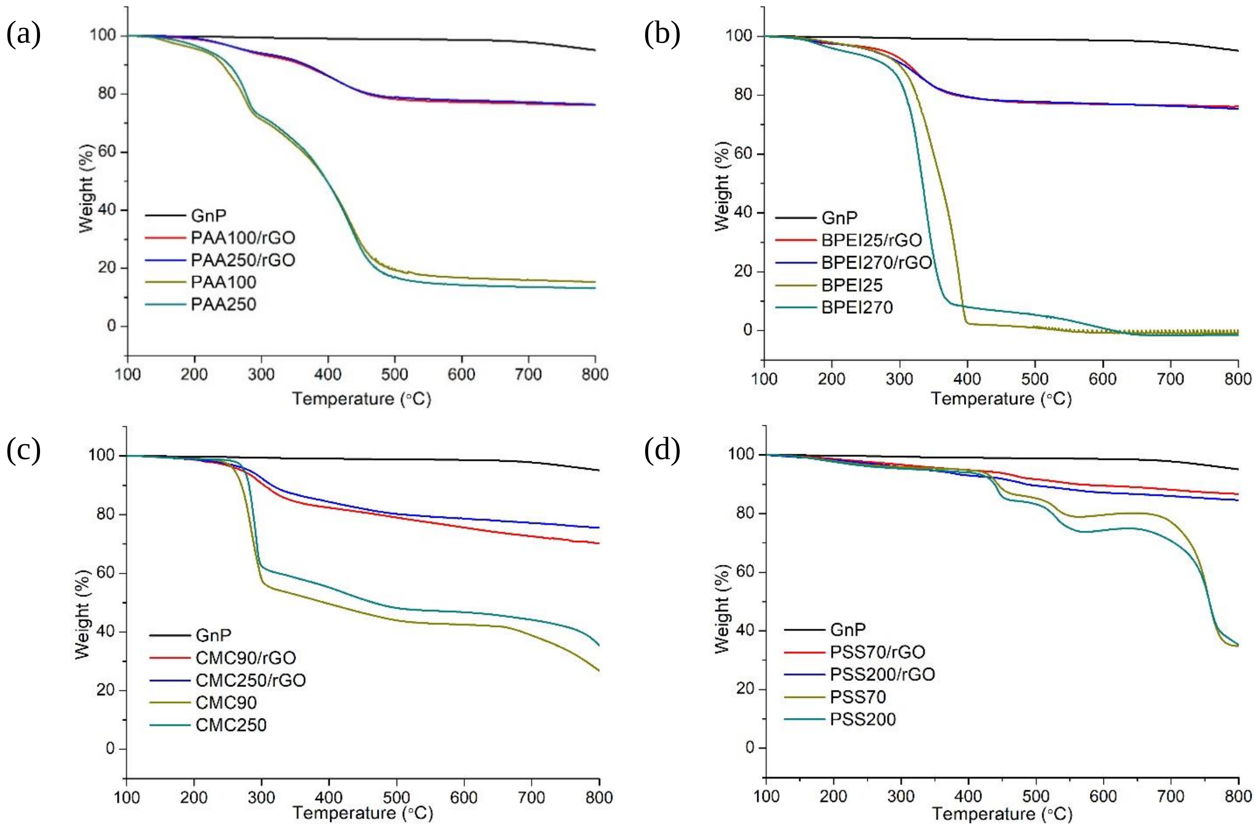

Figure S 5. The TGA curves of PE/rGO after aging 4 weeks.

5. PDI result of samples

Table s 1 The PDI values of PE/rGO samples before and after aging 4 weeks.

PDI	PAA100 /rGO	PAA250 /rGO	BPEI25 /rGO	BPEI270 /rGO	PSS70 /rGO	PSS200 /rGO	CMC90 /rGO	CMC250 /rGO
0 w	0.19	0.32	0.62	0.54	0.60	0.54	0.57	0.46
4 w	0.24	0.25	0.25	0.30	0.50	0.52	0.43	0.41

The Polydispersity index (PDI) was recorded for each sample before and after aging. PAA-based suspensions exhibited the smaller value of PDI corresponding to the narrower particle size distribution and better homogenous dispersion with respect to other PEs. After 4 weeks, the PDI value decreased in most of samples due to the precipitation of large-sized rGO. Among these samples, BPEI-based suspension and PAA-based suspension displayed similar PDI level after 4 weeks. It suggested that a relatively uniform rGO dispersion was obtained after 4-week storing for PAA- and BPEI-based dispersions.

6. Suspensions of PAA100/rGO with different pH

Before sonication, the pH of PAA100 solutions were change by 1M HCl or 1M NaOH. The ionization degree (α) and the dissociation constant (K $_{\alpha}$) represent the dissociation ability of PAA at specific pH, could be calculate via Henderson–Hasselbach equation (3)

$$\mathrm{p}K_A = \mathrm{pH} - \log\frac{\alpha}{1-\alpha} \tag{3}$$

References

[1] U. Khan, A. O'Neill, M. Lotya, S. De, J.N. Coleman, Small 6 (2010) 864-871.

[2] B.P.D. David W. Johnson, Karl S. Coleman Current Opinion in Colloid & Interface Science 20 (2015) 367-382.
