# Supplementary material for: Polyelectrolytes Enabled Reduced Graphite Oxide Water Dispersions: Effects of the Structure, Molecular Weight, and Charge Density
Source: Polymers (Basel). 2022 Oct 4;14(19):4165. doi: 10.3390/polym14194165 (PMC9573485; doi:10.3390/polym14194165)
Supplement: Supplementary file 1 [file polymers-14-04165-s001.zip › Supporting information-videos.pptx]

## Slide 1
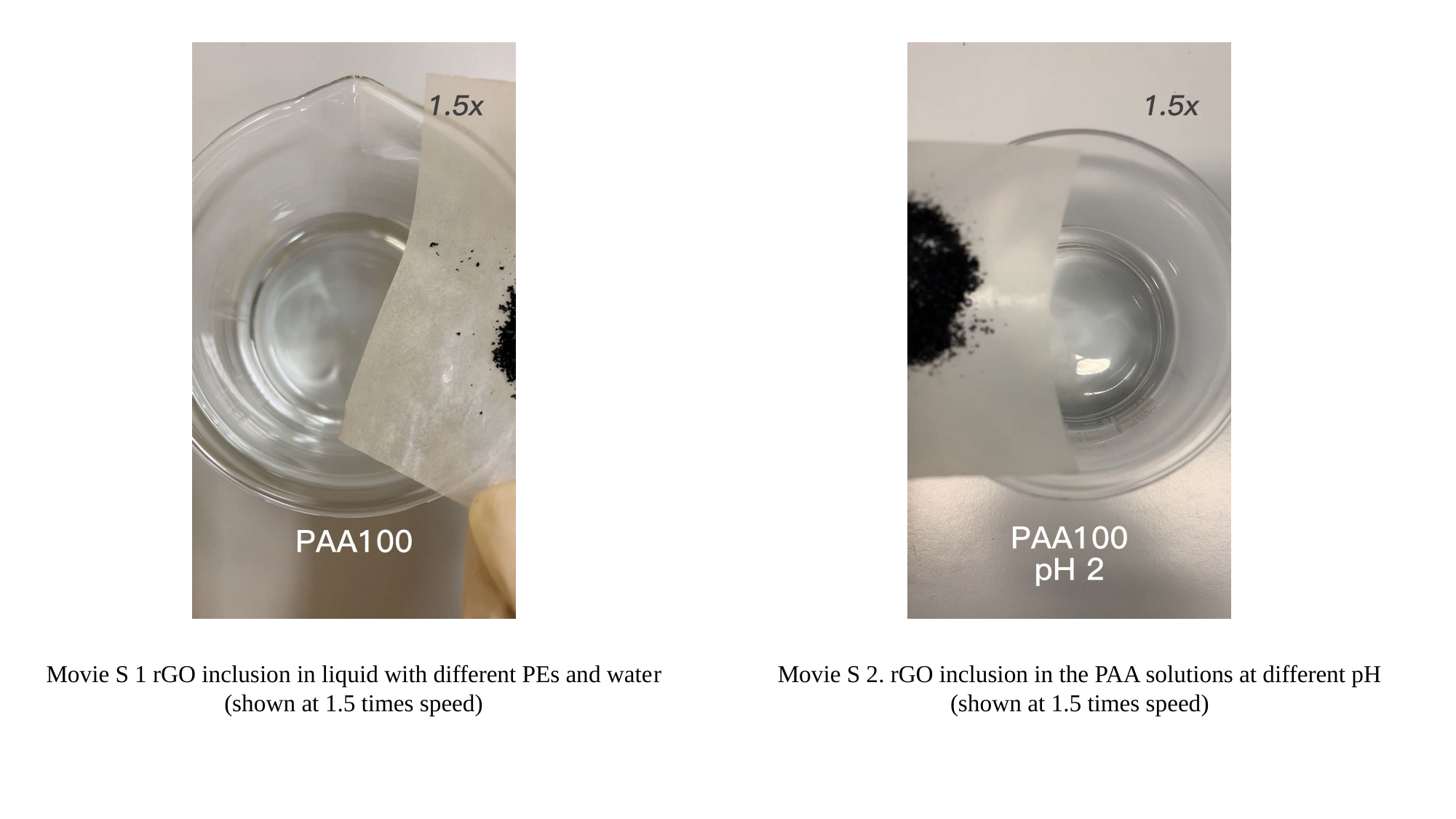

Movie S 2. rGO inclusion in the PAA solutions at different pH (shown at 1.5 times speed)
Movie S 1 rGO inclusion in liquid with different PEs and water (shown at 1.5 times speed)
